# Formation of Thermally Stable Vesicles from a Cyclic Tetraether Archaeal Lipid

**DOI:** 10.1021/acs.langmuir.6c04761

**Published:** 2026-10-21

**Authors:** Valeria Castelletto, Jani Seitsonen, Janne Ruokolainen, Ian W. Hamley

**Affiliations:** † School of Chemistry, Food Biosciences and Pharmacy, 6816 University of Reading, Whiteknights, Reading RG6 6AD, U.K.; ‡ Nanomicroscopy Center, 174277 Aalto University, Puumiehenkuja 2, FIN-02150 Espoo, Finland

## Abstract

The formation of thermostable vesicles by the archaeal lipid β-l-gulopyranosyl-caldarchaetidyl-glycerol (CaldA) is investigated. This caldarchaeol-based lipid comprises the main polar lipid of the archaeon *Thermoplasma acidophilum*. Vesicle formation is shown and quantitatively analyzed through a combination of laser scanning confocal microscopy, cryogenic-transmission electron microscopy (cryo-TEM), dynamic light scattering (DLS), and small-angle X-ray scattering (SAXS). The thermal stability of the CaldA vesicles is then studied after autoclaving. The vesicles are found to be intact after this treatment via cryo-TEM and DLS and fluorescent dye release studies. The limited fluorescence release for CaldA is contrasted with results for the conventional lipid DPPC [1,2-dipalmitoyl-*sn*-glycero-3-phosphocholine], for which significant release of encapsulated calcein is observed on heating. The vesicles are also stable against detergent lysis. Laurdan fluorescence polarization measurements show that the lipid membrane remains in a fluid state across a broad temperature range. The experiments were complemented by atomistic molecular dynamics (MD) simulations which confirm a stable single layer arrangement of the lipids, and also a molecular packing-dependent curvature tendency. The thermostable vesicles of CaldA may be useful for encapsulation applications.

## Graphical abstract

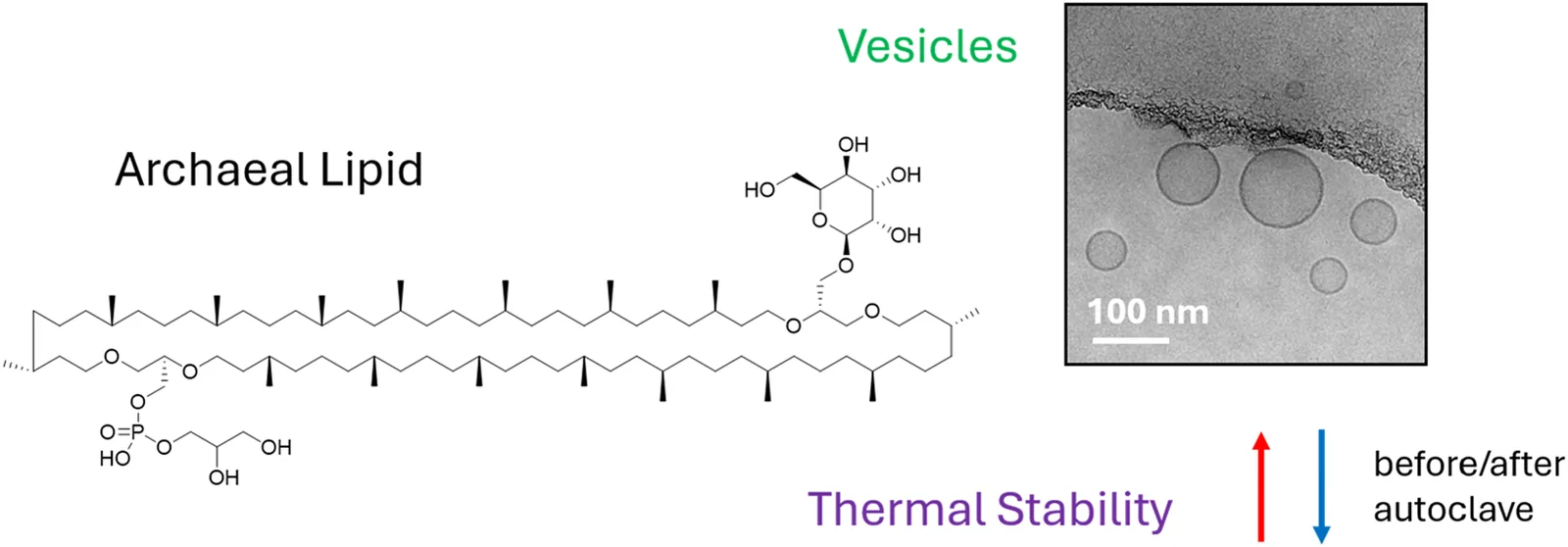

## Introduction

Archaeal organisms are remarkable forms of life that include extremophiles that have evolved in high temperature, high pressure, high salt or high pH conditions, or anaerobic conditions (methanogens). They are found, for example, in deep sea vents or volcano fields. The lipid components of the membranes of archaea have evolved due to their stability in challenging environments, and they are able to self-assemble under such conditions, for example into liposomes (vesicles). Applications include use for encapsulation/slow release drug delivery or as vaccine adjuvants or delivery systems.^1–3^

The composition of lipid membranes has been used as a taxonomic signature to distinguish archaea from bacteria and eukaryotes.^4–6^ Archaeal lipids comprise methylated isoprenoid chains that are linked via ether groups to a glycerol-1-phosphate backbone whereas the phospholipids in bacteria and eukaryotes typically have two fatty acid chains linked by ester groups to a glycerol-3-phosphate group. Archaeal lipids are membrane spanning, i.e., they form monolayer structures rather than the bilayer structure of eukaryote or bacterial membrane phospholipids. An important class of archaeal lipid is the caldarchaeol class which comprises cyclized archaeol isoprenoid chains i.e., C_40_ glycerol-dialkylglycerol tetraethers or glycerol dialkyl glycerol tetrathers (GDGTs), of which one example studied herein is shown in Scheme 1. As well as lipids obtained by extraction from natural archaea, synthetic analogues have been developed and their membrane properties have been examined and exploited for applications.^3,7–9^

**Scheme 1:**
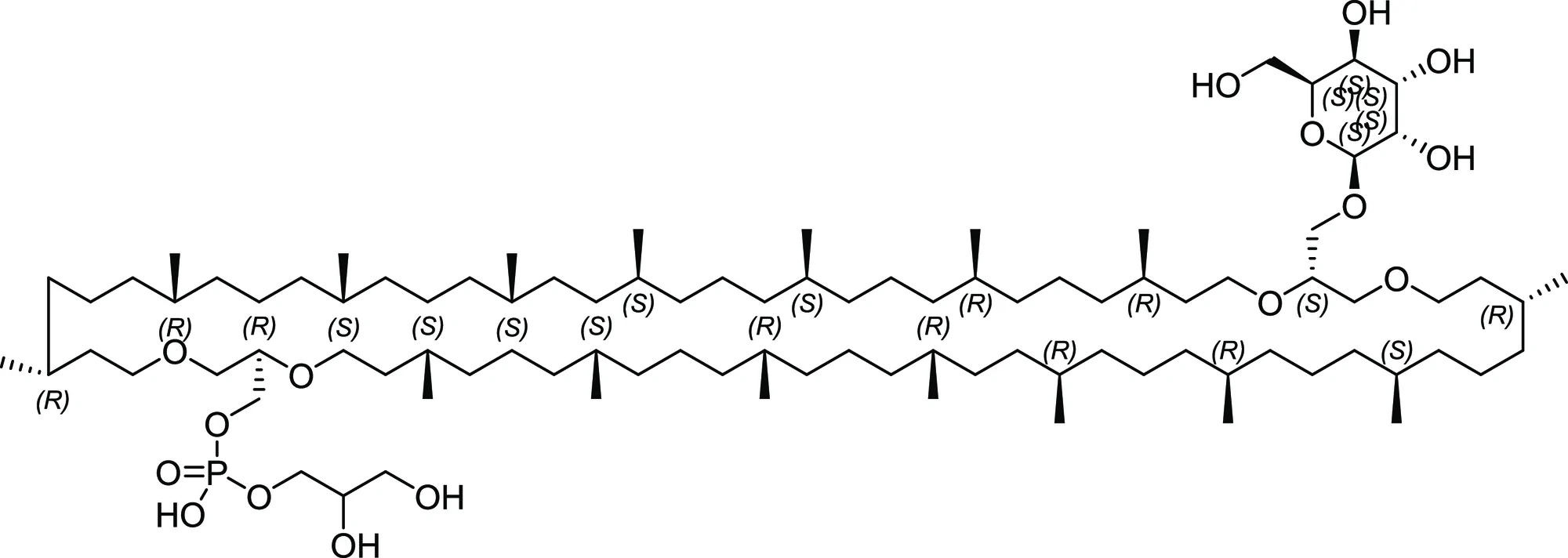
Molecular Structure of β-l-Gulopyranosyl-caldarchaetidyl-glycerol (CaldA).

The thermal stability of liposomes (vesicles) formed by a mixture of caldarchaeol tetraether lipids obtained from the thermophilic archaebacterium *Sulfolobus acidocaldarius* has been investigated.^10^ The major lipid components in the mixture have phosphatidylinositol as one headgroup and either glucopyranose or galactopyranose as the other polar group. The release of carboxyfluorescein from vesicles prepared by extrusion was compared to that of standard phospholipids for comparison. In contrast to POPC (palmitoyl-oleoyl phosphocholine or 1-palmitoyl-2-oleoyl-*sn*-glycero-3-phosphocholine) or DPhPC (1,2-diphytanoylphosphatidylcholine), negligible release of the fluorescent cargo due to vesicle disruption was observed for the archaeal liposomes at 40 or 60 °C, with some limited release at 80 °C observed.^10^ A similar study compared permeability of liposomes from *S. acidocaldarius* tetraether to those from bacterial diester lipids.^11^ In another example, the encapsulation and release of therapeutic compounds from vesicles of a noncyclized synthetic archaea-inspired lipid was studied.^2^

Here, we study the formation of high thermal stability vesicles by the caldarchaeol-based archaeal lipid β-l-gulopyranosyl-caldarchaetidyl-glycerol (thereafter termed CaldA, Scheme 1). This comprises the main polar lipid of the archaeon *Thermoplasma acidophilum*. This primitive organism is found in hot thermal conditions and lacks a cell wall so the lipid membrane is exposed directly to the environment.

We use cryogenic-transmission electron microscopy (cryo-TEM), small-angle X-ray scattering (SAXS), wide angle scattering (WAXS), laser scanning confocal microscopy (LSCM) and dynamic light scattering (DLS) to study the structure of CaldA vesicles. The thermal stability of CaldA vesicles is studied, monitoring the leakage of CaldA vesicles encapsulating 5(6)-carboxyfluorescein (6-CF) or calcein. In particular, the CaldA vesicle calcein release is compared to the leakage from 1,2-dipalmitoyl-*sn*-glycero-3-phosphocholine (DPPC) vesicles. DPPC is chosen as a model lipid to visualize thermally driven vesicle rupture, due to its relatively low gel transition temperature, at 41 °C. Our results show that CaldA vesicles are stable to autoclaving or to treatment with the detergent Triton X-100. Membrane fluidity as a function of temperature is probed using Laurdan fluorescence measurements. The experimental studies of vesicle structure are complemented with atomistic molecular dynamics simulations to probe the ordering in the lipid layer.

## Methods

### Materials

Sample β-l-gulopyranosyl-caldarchaetidyl-glycerol (CaldA, Scheme 1), supplied by Cayman Chemical (USA), was obtained from Cambridge Bioscience (U.K.). Lipid 1,2-sipalmitoyl-*sn*-glycero-3-phosphocholine (DPPC), 5(6)-carboxyfluorescein (6-CF) and Sephadex G-50 were obtained from Sigma-Aldrich (U.K.). Calcein was obtained from TCI (Japan).

#### Sample Preparation

As shown later in the manuscript, CaldA self-assembles into vesicles for all the conditions studied in this work. Therefore, in the following we will refer to ‘vesicle’ preparation for various studies.

##### Preparation of CaldA Vesicles for Structural Studies

For SAXS, WAXS, LSCM and cryo-TEM studies, a stock solution of 0.5 wt % CaldA was prepared by dissolving 1 mg of CaldA, as provided in the form of a dry film by the manufacturer, in 200 μL of Milli-Q water. The solution was mixed by 1 min of alternated vortexing and ultrasound at 55 °C.

##### Encapsulation of 6-CF inside CaldA Vesicles

CaldA was dissolved in choroform and dried under a nitrogen stream to produce a uniform film at the botton of a 1 mL glass vial. The film was resuspended to obtain 0.15 wt % CaldA in 50 mM 6-CF in 10 mM Tris pH 8, and vortexed at 55 °C for ∼1 min, until the lipid was fully dissolved. The resulting solution, containing CaldA vesicles loaded with 6-CF, was run through a Sephadex G-50 gel column as described later in this section. The eluate was diluted to 0.005 wt % CaldA using 130 mM NaCl, 20 mM Tris pH 8.

##### Encapsulation of Calcein inside CaldA Vesicles

CaldA was dissolved in choroform and dried under a nitrogen stream to produce a uniform film at the botton of a 1 mL glass vial. The film was resuspended to obtain 0.16 wt % CaldA in 9.6 mM calcein in 10 mM Tris pH 8, and vortexed at 55 °C for ∼1 min, until the lipid was fully dissolved. The resulting solution, containing CaldA vesicles loaded with calcein, was run through a Sephadex G-50 gel column as described later in this section. The eluate was diluted to 0.01 wt % CaldA using 130 mM NaCl, 20 mM Tris pH 8.

##### Ecapsulation of Calcein inside DPPC Liposomes

An aliquot of DPPC was dissolved in chloroform inside a 1 mL glass vial. The choroform was evaporated under a nitrogen stream, to produce a lipid film at the bottom of the flask. The film was then resuspended by adding a solution containing 2.5 mM calcein and 14 mM NaOH, and vortexing at 65 °C for 15 s. The produced 0.15 wt % DPPC liposomes loaded with 2.5 mM calcein, were then run through a Sephadex G-50 gel column, to remove the excess of calcein.

##### Preparation and Use of Sephadex G-50 Gel Column

Solutions containing CaldA vesicles loaded with 6-CF or calcein were run thorugh a Sephadex G-50 gel column, using gravity, to extract the excess dye from the solvent. To prepare the column, 200 mg of Sephadex G-50 was put in a glass vial and covered with 2 mL of water. The mixture was left to stabilize and form a soft gel. A Pasteur pipet was used to build a column for the gel. A small cotton ball was placed at the narrow end of the pipet, to filter spurious particles from the final product, while the narrow tip of the pipet was shortened to facilitate the release of the filtered solution. The tip of a 1 mL plastic pipet was cut, before using it to put the gel inside the glass Pasteur pipet, and build a 3 cm high column of gel. Before use, the gel column was rinsed and stabilized with the same buffer used to prepare the corresponding CaldA vesicle solution.

Dynamic light scattering (DLS) was used to verify the successful separation of the vesicles loaded with dye from free dye molecules in solution. As discussed later in the manuscript, the hydrodynamic radius, *R*_H_, measured for CaldA vesicles encapsulating 6-CF or calcein (SI Figure S3 and Table S1) is the same as *R*_H_ measured for CaldA vesicles in water (Figure 4 and Figure 2 and SI Table S1).

#### Autoclaving

An aliquot 1 mL of a solution containing CaldA vesicles was placed in a 1.5 mL glasss vial with a loose fitting cap. The container was placed inside the chamber of a Priorclave Front Loading autoclave instrument, before starting the autoclave cycle. The solution was then autoclaved using a liquid cycle with a low exhaust which included boiling at 115 °C (+0.7 bar) for 10 min. Following the cycle, the sample was allowed to return to 20 °C before being used for DLS or fluorescence experiments explained later in the manuscript.

#### Fluorescence Spectroscopy

Vesicle stability was studied using fluorescence spectroscopy. The fluorescence emission of dye leakage from CaldA vesicles loaded with 6-CF or calcein and from DPPC liposomes loaded with calcein, was measured using a Varian Model Cary Eclipse spectrofluorometer. Solutions were loaded in a 10 mm light path quartz cell. Excitation wavelengths λ_ex_ = 492 or 486 nm were used to measure the emission fluorescence from vesicles loaded with 6-CF or calcein, respectively.

The emission fluorescence of CaldA vesicles loaded with 6-CF or calcein and the emission fluorescence of DPPC liposomes loaded with calcein was measured as a function of the temperature for *T* = 20–80 °C. Thereafter, the fluorescence of the same samples, but treated with 0.1 wt % Triton X-100, was measured as a function of the temperature for *T* = 20–60 °C. Samples treated with 0.1% Triton X-100 were cloudy at *T* = 70–80 °C and therefore were not studied by fluorescence.^12^

Data collected from the experiments described in the paragraph above was used to estimate the release of fluorophore from DPPC vesicles accordying to

1 $$\%\mathrm{release}:\;100\times \left[\left(I_{T}-I_{o}\right)/\left(I_{L}-I_{o}\right)\right]$$

where *I_T_* is the fluorescence intensity of the sample at temperature *T*, *I*_o_ is the fluorescence intensity of the sample at 20 °C, and *I*_L_ is the fluorescence intensity of the sample at temperature *T* measured after complete lysis of the vesicles using Triton X-100.

In a separate experiment, CaldA vesicles loaded with calcein or 6-CF were autoclaved before measuring the fluorescence emission of the solution at 20 °C.

#### Cryo-Transmission Electron Microscopy (Cryo-TEM)

Imaging was carried out using a field emission cryo-electron microscope (JEOL JEM-3200FSC), operating at 200 kV. Images were taken in bright field mode and using zero loss energy filtering (omega type) with a slit width of 20 eV. Micrographs were recorded using a Gatan Ultrascan 4000 CCD camera. The specimen temperature was maintained at −187 °C during the imaging. Vitrified specimens were prepared using an automated FEI Vitrobot device using Quantifoil 3.5/1 holey carbon copper grids with a hole size of 3.5 μm. Just prior to use, grids were plasma cleaned using a Gatan Solarus 9500 plasma cleaner and then transferred into the environmental chamber of a FEI Vitrobot at room temperature and 100% humidity. Thereafter 3 μL of sample solution was applied on the grid and it was blotted twice for 5 s and then vitrified in a 1/1 mixture of liquid ethane and propane at temperature of −180 °C. The grids with vitrified sample solution were maintained at liquid nitrogen temperature and then cryo-transferred to the microscope.

#### Small Angle (SAXS) and Wide Angle (WAXS) X-ray Scattering

Experiments were performed on beamline B21 ^13^ at Diamond (Didcot, UK). The sample solutions were loaded into the 96-well plate of an EMBL BioSAXS robot and then injected via an automated sample exchanger into a quartz capillary (1.8 mm internal diameter) in the X-ray beam. The quartz capillary was enclosed in a vacuum chamber, to avoid parasitic scattering. After the sample was injected into the capillary and reached the X-ray beam, the flow was stopped during the SAXS data acquisition. Beamline B21 operates with a fixed camera length (3.9 m) and fixed energy (12.4 keV). The images were captured using a PILATUS 2 M detector. The WAXS data was acquired using a Dectris Eiger 2 1 M detector, and the *q*-axis was calibrated using the diffraction spectrum of silver behenate and the SAXS intensity was normalized using the signal of water. Data processing was performed using dedicated beamline software ScÅtter.

#### Dynamic Light Scattering (DLS)

Experiments were performed using an ALV CGS-3 system with 5003 multidigital correlator. The light source was a 20 mW He–Ne laser, linearly polarized, with λ = 633 nm. A scattering angle θ = 90° was used for all the experiments. Samples were loaded into standard 0.5 cm diameter cylindrical glass cells. Solutions were filtered using inorganic membrane filters, Whatman Anotop 10 mm diameter, 0.2 μm pore size, before experiments. The intensity autocorrelation functions were analyzed by the constrained regularized CONTIN method^14^ to obtain distributions of hydrodynamic radius, *R*_H_. The error Δ*R*_H_ was estimated as half width at half intensity of the corresponding peak in the hydrodynamic radius distribution function.

#### Laser Scanning Confocal Microscopy (LSCM)

Samples containing 0.5 wt % CaldA were stained with Rhodamine B (RhoB; highly hydrophilic, zwitterionic in water). Since RhoB was added to the CaldA solution rather than encapsulated in the vesicles, it is expected that it will bind by electrostatic interaction to the surface of the vesicles

LSCM experiments were run with a solution containing 0.25 wt % CaldA in 3 × 10^–4^ wt % RhoB, using a Nikon A1R confocal microscope. Solutions were loaded in a chamber slide with 8 wells on a glass slide. Experiments were performed using a Plan Apo λ 100× Oil lens, and pinhole size 20.43 μm. The sample was excited at λ_ex_ = 561 nm while the emission was measured at λ_em_ = 595 nm. The microscope was also used to record images in bright field transmission mode.

#### Laurdan Fluorescence

An aliquot 200 ul of a solution containing 0.3 wt % CaldA dissolved in 0.01 wt % Laurdan in chloroform were placed in a 1 mL glass vial. The chloroform was evaporated from the solution under a Nitrogen stream to create a film at the bottom of the flask. Enough water to reach 0.05 wt % CaldA in 2 × 10^–3^ wt % Laurdan was added to the flask. The film was resustended by vortexing the solution at 55 °C for ∼1 min. The solution was left to rest for 30 min and diluted 5-fold in water, to provide 0.01 wt % CaldA in 5 × 10^–4^ wt % Laurdan. The Laurdan fluorescence excitation spectrum was measured using λ_em_ = 440 nm. The fluorescence emission spectrum was measured as a function of temperature, for 20 °C ≤ *T* ≤ 90 °C, using λ_ex_ = 356 nm, as determined from the fluorescence excitation spectra. In a disordered lipid environment Laurdan’s emission maximun is centered at 490 nm while in more ordered lipid sourroundings it shifts to 440 nm. Hence, the lipid packing in the bilayer can be qualitatively expressed by the Laurdan generalized polarization parameter^15,16^

2 $$g\left(T\right)=\left(I_{440}-I_{490}\right)/\left(I_{440}+I_{490}\right)$$

where, *I*_440_ and *I*_490_ are the intensity of the fluorescence emission at 440 and 490 nm, respectively. The parameter *g*(*T*), calculated using eq 2, was used to determine the conformation of the lipidic chains as a function of the temperature.

#### Molecular Dynamics Simulations

Molecular dynamics simulations were performed using Gromacs^17^ (version 2024.4). The force field parameters for CaldA were obtained using the CHARMM-GUI ligand reader and modeler.^18,19^ Simulations were performed using the CHARMM36 force field.^20,21^ An initial configuration of CaldA with extended chains was generated manually and molecular mechanics energy minimization was performed leading to the structure shown in SI Figure S9a. A single layer comprising 300-500 of these molecules (with random in-plane order) was constructed using Packmol.^22^ The simulated systems are listed in SI Table S3. The packed molecules were placed into a cubic box of dimensions 20 × 20 × 20 nm^3^, and the system was solvated using TIP3P water. After energy minimization and 100 ps relaxation stages in the NVT ensemble, the final simulations were carried out in the NPT ensemble using a leapfrog integrator with steps of 1 fs up to 20 or 40 ns (20,000,000 or 40,000,000 steps). The temperature was maintained at 298.15 K using the velocity-rescale (modified Berendsen) thermostat^23^ with a time constant of 5 ps. The pressure was maintained at 1 bar using the Parinello-Rahman barostat^24^ (isotropic pressure coupling) and periodic boundary conditions were applied in all three dimensions. The Particle Mesh Ewald scheme^25,26^ was used for long-range electrostatics. Bonds were constrained using the LINCS algorithm^27^ and the Verlet cutoff scheme^28^ was used. Coulomb and van der Waals cutoffs were 1.0 nm.

## Results and Discussion

We investigated the self-assembly of the caldarchaeol cyclic tetraether lipid β-L-gulopyranosyl-caldarchaetidyl-glycerol (Scheme 1, hereafter termed CaldA), a type of archaeal lipid, and its use as thermally stable vesicular release system. A combination of cryo-TEM, dynamic light scattering (DLS) and X-ray scattering (SAXS/WAXS) were used to characterize the formation of vesicles (liposomes) by CaldA. The representative cryo-TEM image in Figure 1a confirms that CaldA forms well-defined vesicles with thin walls and a distribution of sizes. DLS was used to determine the sample-averaged distribution of vesicle sizes. The corresponding hydrodynamic radii are listed in SI Table S1. Confocal microscopy (LSCM) images for samples stained with rhodamine B (SI Figure S1) show the presence of a population of larger vesicles in addition to those imaged by cryo-TEM and sized by DLS.

**Figure 1:**
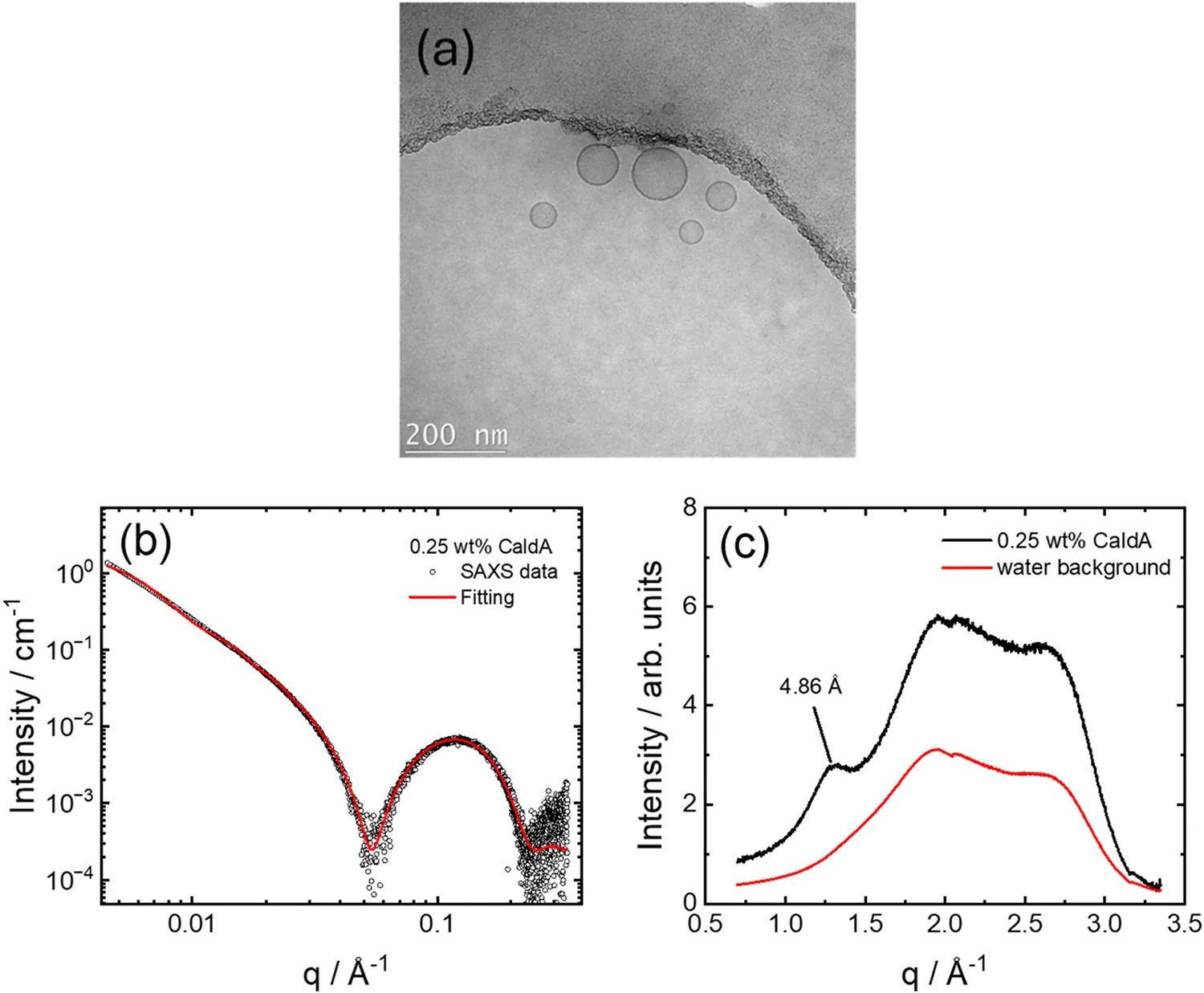
(a) Cryo-TEM images showing vesicles for a 0.25 wt % CaldA solution. (b) SAXS and (c) WAXS data for 0.25 wt % CaldA. The full lines in (b) are the fitting to the SAXS data as described in the manuscript (fit parameters in SI Table S2). Data in (c) is scaled for ease of visualization.

The structure of the vesicle membrane was probed using simultaneous small-angle X-ray scattering/wide-angle X-ray scattering (SAXS/WAXS). The data are shown in Figure 1b,c. The SAXS data (Figure 1b) shows the form factor of unilamellar vesicles. There are no Bragg peaks associated with the structure factor of multilamellar vesicles. The data are very well described by a model developed for conventional lipid membranes in which the electron density profile across the membrane is represented as sum of three Gaussian functions, two representing the two exterior headgroups and one representing the lower electron density of the hydrophobic interior.^29^ We recently use this model to represent the form factor of unilamellar vesicles formed by Toll-like receptor agonist lipidated glyceryl-cysteine conjugates.^30^ Here, the SAXS data in Figure 1b are well described by the Gaussian bilayer model. The fit parameters listed in SI Table S2 show that the bilayer thickness is *t* = (41.02 ± 0.58) Å. This is close to the estimated molecular length (Scheme 1) for 36 carbons in an extended linear stretch of the cyclic molecule and indicates that the membrane has a single layer structure with the hydrated glycerol groups at the two water interfaces and with a hydrophobic isoprenoid lipid interior. This analysis was used as input for the MD simulations discussed below. The WAXS data presented in Figure 1c shows that the lipid chains are well ordered with a WAXS peak at *d* = 4.86 Å, the average interchain spacing.

Having established that CaldA self-assemble into vesicles in solution, we then proceeded to study the stability of the vesicles as a function of the temperature or against lysis induced by Triton X-100. DLS measurements were used to calculate the distributions of hydrodynamic radii, *R*_H_, for 0.01 wt % CaldA vesicles at 20 °C, cooled to 20 °C after autoclaving, or after heating to 65 °C. The results are displayed in Figure 2. Values of hydrodynamic radius measured from the distributions in Figure 2 are listed in SI Table S1.

**Figure 2:**
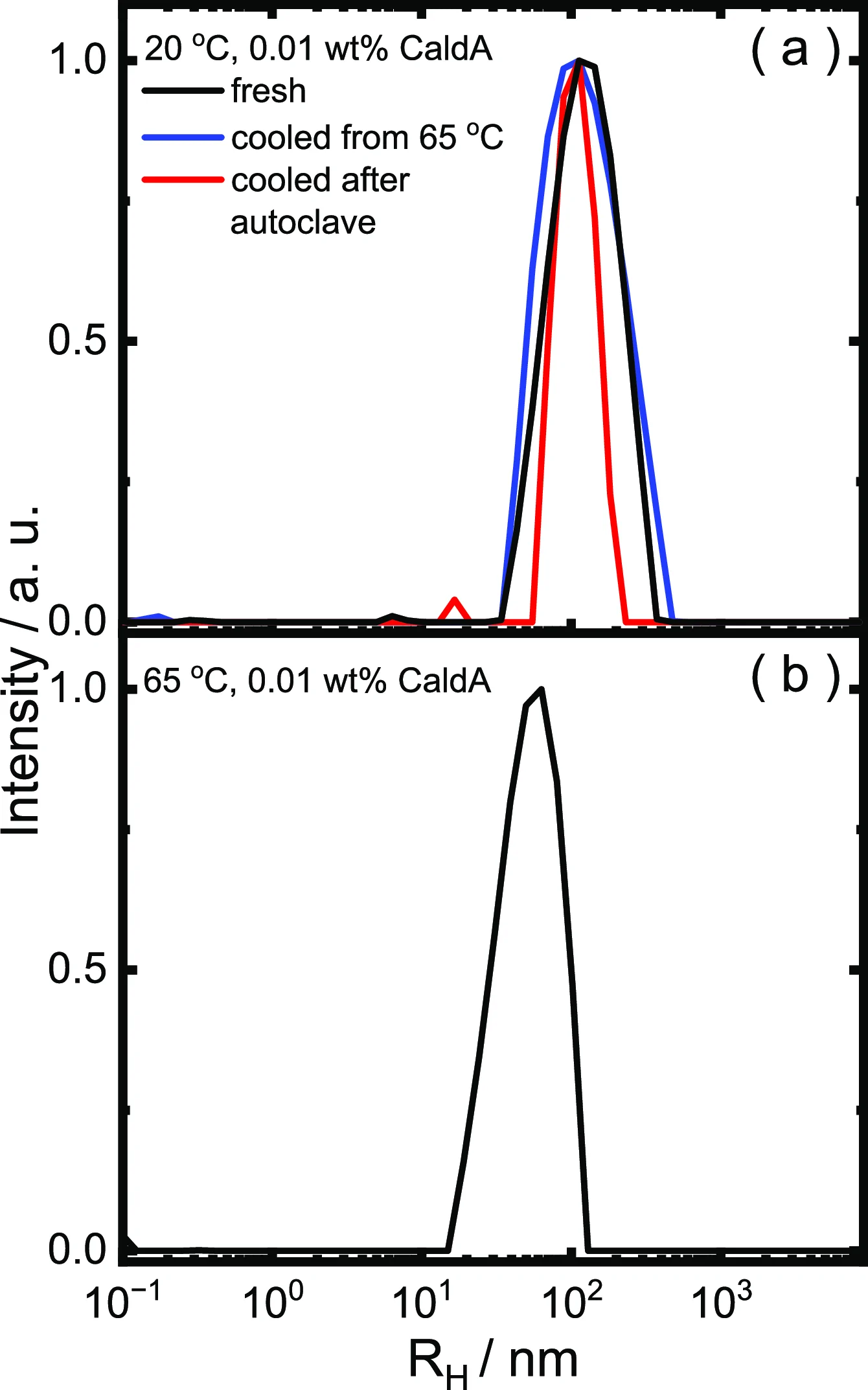
Distribution of hydrodynamic radius, *R*_H,_ for 0.01 wt % CaldA. (a) Data at 20 °C: fresh solution or solution cooled to 20 °C after autoclaving or from 65 °C; (b) solution at 65 °C.

The distribution of hydrodynamic radius in Figure 2a presents a primary peak at 20 °C corresponding to *R*_H_ = 113 nm. Remarkably, this does not change substantially if the solution is cooled to 20 °C after autoclaving for 10 min at 115 °C (Figure 2a,b and Table S1). The hydrodynamic radius decreases upon heating, for example to *R*_H_ = 63 nm at T= 65 °C (Figure 2b and SI Table S1).

The *R*_H_ distributions in Figure 2 do not show sizes corresponding to the larger vesicles imaged by LSCM (SI Figure S1) because, unlike LSCM samples, DLS solutions were filtered before the experiment.

Having shown that heating or even autoclaving the sample does not substantially affect the size of the CaldA vesicles, we studied the stability of the vesicles by emission fluorescence assays. For these experiments, two different dyes, 5(6)-carboxyfluorescein or calcein, were encapsulated in CaldA vesicles as described in the Experimental Section. CaldA vesicles loaded with dye were exposed to different thermal conditions or a lysing agent, to break the wall of the vesicles and allow for the release of the encapsulated dye. The data in Figure 3a,b (original spectra in SI Figure S2) shows the emission fluorescence intensity of CaldA vesicles heated from 20 to 80 °C. Following the temperature ramp, the solution was either autoclaved for 10 min at 115 °C and 0.7 bar or treated with Triton X-100 (a detergent with lysis activity).

**Figure 3:**
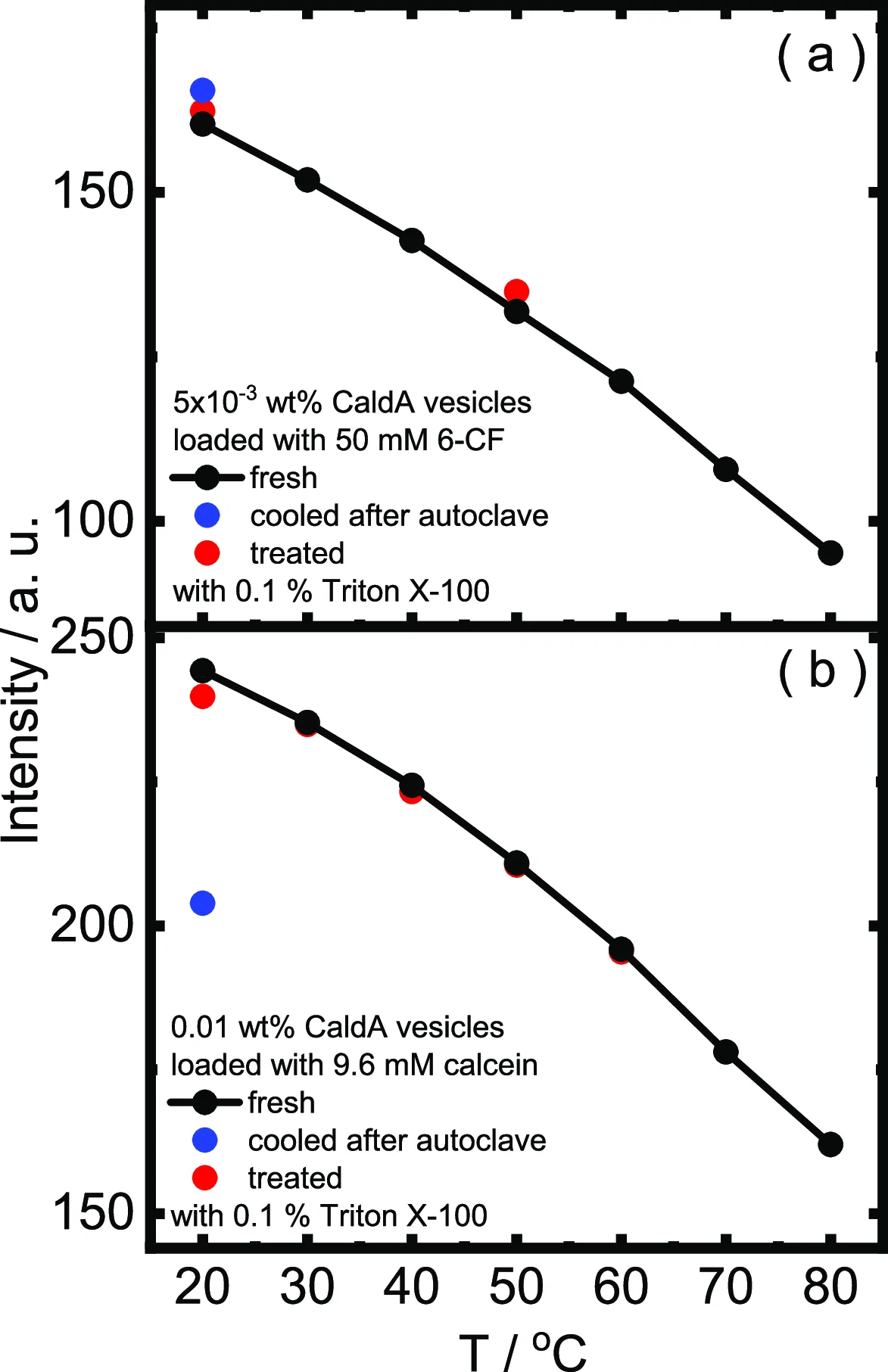
Maximum fluorescence emission intensity measured for CaldA vesicles loaded with (a) 6-CF or (b) calcein for different conditions, as indicated in the figure caption: fresh solution, solution cooled after autoclaving or solution treated with 0.1 wt % Triton X-100.

The data in Figure 3 show no increase in the fluorescence emission due to vesicle breakage, when CaldA vesicles are exposed to extreme temperatures or Triton X-100. In fact, the peak emission fluorescence intensity decreases with increasing *T*. It is possible that such a quenching effect may be caused by a reduction in size of the vesicle with increasing T. Indeed, the DLS data in Figure 2 (SI Table S1) shows that *R*_H_ decreases upon increasing temperature from 20 to 65 °C.

To better understand these results, *R*_H_ was measured for a fresh sample and the sample corresponding to autoclaved vesicles that provided the data in Figure 3. The size distributions in SI Figure S3 show first that loading of the dyes does not significantly affect the vesicle radius. In addition, autoclaving does not affect the *R*_H_ value of CaldA vesicles loaded with dye, i.e., boiling does induce any leakage of encapsulated dye. The stability of vesicles after autoclaving was also confirmed by cryo-TEM images of intact vesicles, of which a representative example is shown in SI Figure S4.

These results highlight the remarkable resistance of CaldA vesicles to heat and detergent lysis, and they are in contrast with the results expected for vesicles made of model lipids, such as DPPC. This was confirmed by studying the thermally induced breakage of DPPC vesicles loaded with calcein. The maximum fluorescence emission intensity measured as a function of the temperature for DPPC vesicles loaded with calcein is shown in SI Figure S5a (original spectra in SI Figure S6), using a fresh solution and a solution treated with 0.1 wt % Triton X-100. SI Figure S5b shows the percentage of calcein released from DPPC vesicles, calculated using eq 1. It is evident that increasing the temperature breaks the walls of DPPC vesicles allowing for the release of the calcein entrapped inside the vesicles.

The properties of the CaldA bilayers were further studied by Laurdan generalized polarization fluorescence experiments. Laurdan is a fluorescent probe, highly sensitive to water penetration in lipid bilayers and commonly used to study the fluidity of lipid membranes.^31^ In this way, the fluidity of the CaldA membrane as a function of *T* was examined. SI Figure S7 displays the Laurdan emission fluorescence curves for 0.01 wt % CaldA vesicles loaded with 5 × 10^–4^ wt % Laurdan. The data in SI Figure S7 was used to calculate the generalized polarization factor g­(T) according to eq 2 and the results are shown in Figure 4. Laurdan undergoes a red spectral shift during the lipid membrane phase transition from gel to fluid, which is attributed to dipole relaxation and the sensitivity to the polarity of the environment.^15,32^ Consequently, a Laurdan probe placed in a low polarity (lipid bilayer in the gel phase) or high polarity (lipid bilayer in the fluid phase) environment is characterized by values of *g*(*T*) in the range 0.6–0.8 or *g*(*T*) ∼ [(−0.4) – (−0.2)] respectively.^15,32^ The data in Figure 4 show that there is not a defined melting temperature for the CaldA chains in the vesicle walls, rather the lipid membrane is in the fluid state for the whole range of temperatures. At first sight, this result contradicts the low water permeability attributed to archaeal lipids.^33^ However, it has been demonstrated that when interacting with archaeal lipid membranes, the Laurdan headgroup anchors to the surface of the membrane, remaining highly exposed to water, while the Laurdan hydrocarbon tail penetrates parallel to the hydrophobic region of the membrane. This configuration produces negative *g*(*T*) values.^34^ Laurdan generalized polarization factors with similar values and smooth *T*-dependence have been reported for closely related lipids.^34–36^ Lipid phase transition temperatures for CaldA are not reported in the literature, to the best of our knowledge. Analogous archaeal polar lipid membranes can be presumed to be in a liquid crystalline phase in the temperature range of 0 to 100 °C.^37^

**Figure 4:**
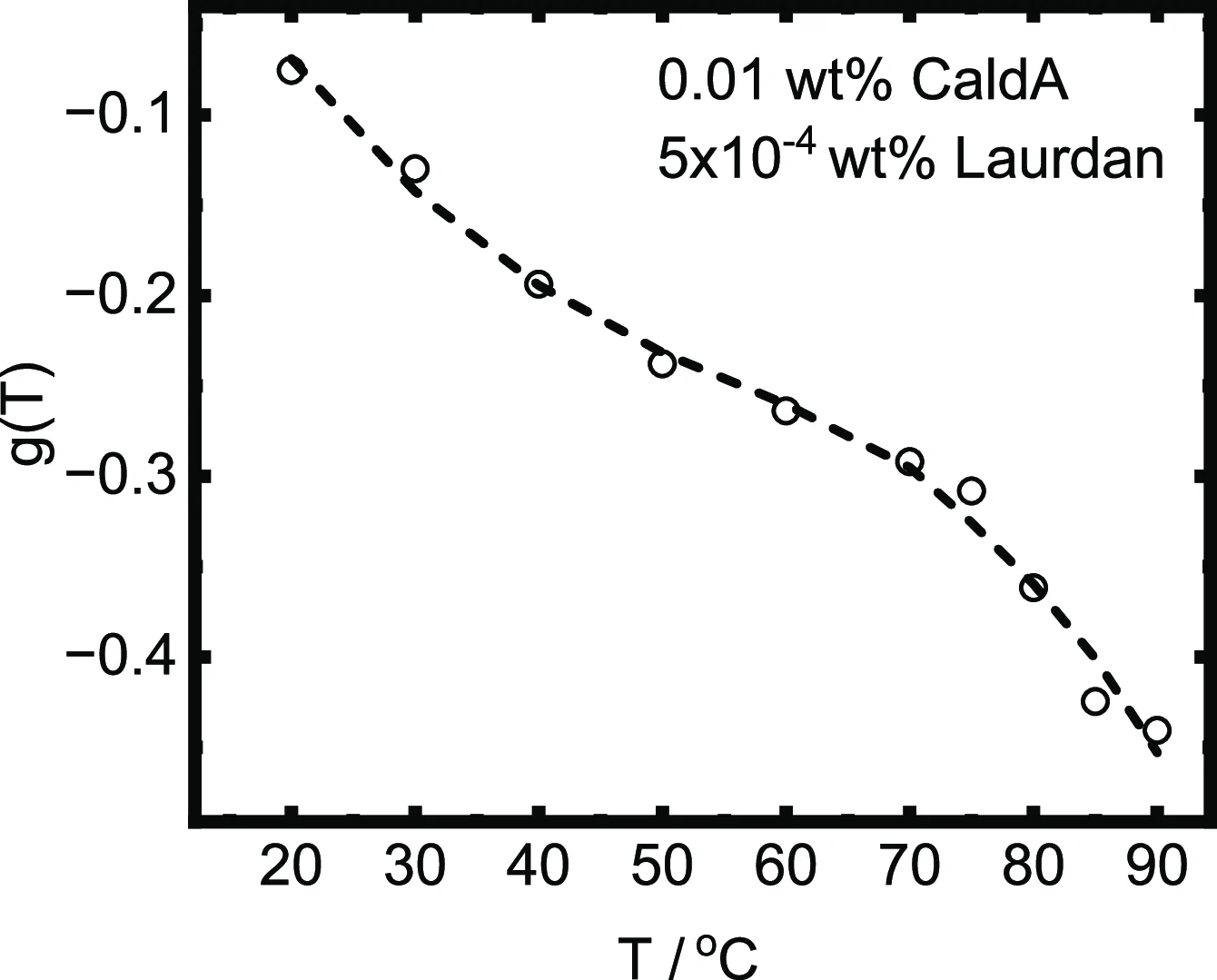
Laurdan generalized polarization factor calculated for 0.01 wt % CaldA vesicles loaded with 5 × 10^–4^ wt % Laurdan.

The experimental studies of vesicles were complemented by atomistic molecular dynamics (MD) simulations to probe membrane curvature and molecular conformation within the vesicle wall. Based on the SAXS data which shows a single layer thickness, a single layer was constructed in a water box. We explored the effect of packing density and packing asymmetry by performing simulations for monolayer structures and fully interdigitated bilayers with the same single layer thickness. The simulations performed are listed in SI Table S3 and images from the final frames of simulations are presented in SI Figure S8. A representative initial configuration is shown in SI Figure S9. For all simulations, the layer structure was found to be stable during extended simulation runs. SI Movie S1 shows the structure from the final frame of an extended run for system A in SI Table S3. Representative images of the structure for system A after equilibration are shown in Figure 5(a,b). Equilibration was confirmed during the simulation run by monitoring the solvent-accessible surface area (SASA) and related properties shown in SI Figure S10, which plateau after around 20 ns. The density profile across the layer shown in Figure 5c shows the expected symmetric maximum with FWHM 3.45 nm, which is in good agreement with the layer thickness *t* from SAXS (SI Table S2). The maxima associated with the oxygen atoms in the gulose or phosphoglycerol saccharide units (Scheme S1) are also evident in Figure 5c, and as expected are located away on either side of the layer center plane.

**Figure 5:**
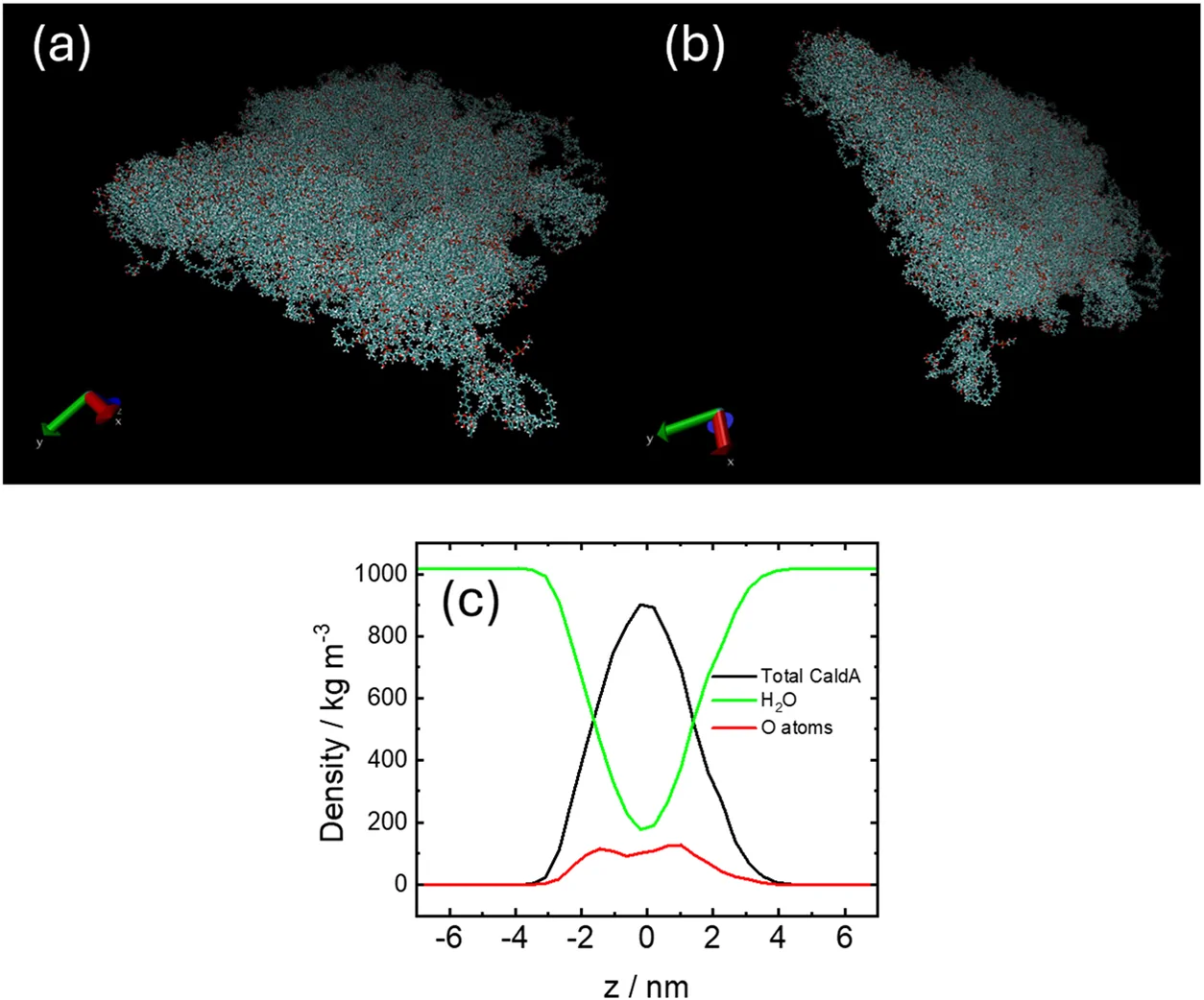
(a, b) Two projections of monolayer of CaldA from final frame of MD simulations of system A (SI Table S3), (c) density profiles (from last 500 ps of 40 ns simulation run) across the monolayer showing total lipid density as well as density of water and oxygen atoms associated with the saccharide groups.

Comparing different simulated systems (SI Table S3), it was noted that the packing density within the layer influenced the tendency of the layer to exhibit spontaneous curvature. The preceding results showing a stable nearly flat layer were obtained with system A in SI Table S3 (at a density of 10.2 molecules nm^–2^). With a lower packing density, (System B, 3.5 molecules nm^–2^) significant spontaneous membrane curvature of the layer was observed. This is shown by representative images from the final frame of MD simulations in SI Figure S11. SI Movie S2 shows the membrane curvature starting from the initial planar configuration (which was already observed during the canonical phase of the simulation) and SI Movie S3 is a rotation movie to more clearly show the positive Gaussian curvature. As shown in SI Figure S8, curvature was also observed in simulations for fully interdigitated bilayers (systems D and E, SI Table S3). These results suggest that spontaneous curvature into vesicles can occur depending on the molecular packing and density in the layer, although edge effects from the finite size of the layers in the simulation may also play a role.

## Conclusions

In summary, CaldA self-assembles into vesicles, and we have comprehensively studied the size and structure of the liposomes across length scales. SAXS shows that the lipid membrane is a layer with thickness 41 Å, while the lipid ordering is characterized by a *d* = 4.86 Å WAXS peak.

The thermostability of the CaldA vesicles was probed by DLS as well as fluorescence studies of release of the dyes 5(6)-carboxyfluorescein and calcein. The size distribution from DLS shows a characteristic vesicle diameter which is retained after heating or autoclaving. No increase of fluorescence of the encapsulated fluorescent dyes was observed upon heating to 80 °C, nor after autoclaving, nor in the presence of Triton X-100. The latter points to the high stability of the vesicles in the presence of detergents, and additional feature to high thermal stability. These results contrast with the significant release observed for conventional DPPC bilayer phospholipid vesicles.

The ordering of the CaldA lipids in the membrane was probed using Laurdan generalized polarization measurements which show fluid-like properties across the temperature range studied (20–90 °C).

The experimental studies were complemented with atomistic MD simulations which demonstrated the stability of single layer packings of CaldA, the obtained layer thickness being consistent with that obtained from fitting of SAXS data. Spontaneous curvature of the membrane was observed upon lowering the molecular packing density. The curvature presumably arises from the asymmetry in molecular packing at the inner and outer membrane walls, although edge effects may also have an effect, considering the finite size layers simulated. These phenomena require future examination via further simulations and/or theoretical analysis.^38,39^

The formation of single layer vesicles by the archaeal lipid contrasts with the formation of bilayer vesicles by phospholipids but the high thermal stability may reflect the origins of archaea species. The CaldA liposomes may have applications in formulation where high thermal stability encapsulation is desired, for example for specialist cleaning products or drug delivery.^40–42^ The lipid monolayers may also have other uses such as thermally stable membranes for separation.

## Supplementary Material









## Notes

Cryo-TEM image histograms, additional LSCM images, and size distribution, original fluorescence spectra, fluorescence spectra and release data for DPPC vesicles, snapshots of structures from MD simulations, plot of solvent-accessible surface area and related parameters, Tables of SAXS fit parameters (DOCX)
Rock/roll image of final frame from full simulation run showing flat membrane (Movie S1) (MPG)
Trajectory showing transition of membrane from planar to positive Gaussian curvature (from initial nvt simulation) from a simulation with lower packing density (Movie S2) (MPG)
Rock/roll image of final frame from full simulation run showing membrane curvature from a simulation with lower packing density (Movie S3) (MPG)
