# Supplementary material for: Formation of Thermally Stable Vesicles from a Cyclic Tetraether Archaeal Lipid
Source: Langmuir. 2026 Oct 21;42(43):32101–8. doi: 10.1021/acs.langmuir.6c04761 (PMC13738793; doi:10.1021/acs.langmuir.6c04761)
Supplement: Supplementary file 1 [file la6c04761_si_001.docx]

**Supporting Information**

**Formation of Thermally Stable Vesicles from a Cyclic Tetraether Archaeal Lipid**

Valeria Castelletto, ^1^ Jani Seitsonen,^2^ Janne Ruokolainen,^2^ Ian W Hamley^1,*^

*^1^ School of Chemistry, Food Biosciences and Pharmacy, University of Reading, Whiteknights, Reading RG6 6AD, U.K.*

*^2^ Nanomicroscopy Center, Aalto University, Puumiehenkuja 2, FIN-02150 Espoo, Finland*

* Author for correspondence, I.W.Hamley@reading.ac.uk

**SI Fig.S1** LSCM image of vesicles formed by 0.25 wt% CaldA stained with 3×10^-4^ wt% RhoB.

**SI Fig.S2.** Fluorescence emission curves of CaldA vesicles loaded with (a,b) 6-CF or (c,d) calcein. Conditions as indicated in the figure caption.

**SI Fig.S3.** Distribution of hydrodynamic radius, R_H_, for CaldA vesicles used in Fig 3. Data for vesicles loaded with (a) 6-CF or (b) calcein, measured using a fresh or a solution cooled after autoclaving.

**SI Fig.S4**. Cryo-TEM image of 0.01 wt% solution of CaldA after boiling, showing intact vesicles.

**SI Fig S5**. (a) Maximum fluorescence emission intensity measured for DPPC vesicles loaded with calcein for the fresh solution and the solution treated with 0.1 wt% Triton X-100. (b) Percentage calcein released by the vesicles calculated using data in (a).

**SI Fig S6.** Fluorescence emission curves of DPPC vesicles loaded with calcein (a) before and (b) after lysis induced by Triton X-100 treatment. Conditions as indicated in the figure caption.

**SI Fig S7.** Laurdan emission fluorescence spectra as a function of the temperature, measured for CaldA vesicles loaded with Laurdan.

**SI Fig.S8.** Images from final frame of MD simulations for the systems listed in SI Table S3.

**SI Fig.S9.** Initial structures for MD simulations, (a) CaldA molecule, (b) CaldA monolayer in box (filled with TIP3P water molecules, not shown for clarity).

**SI Fig.S10.** Solvent-accessible surface area (SASA) and related properties from MD simulations on a CaldA monolayer, system A from SI Table S3 (pairs of lines represent data from two runs, one of 20 ns, the other of 40 ns).

**SI Fig.S11**. Spontaneously curved membrane observed in MD simulations on system B. Two projections of monolayer of CaldA from final frame of MD simulations described in the text.

**SI Table S1.** Average hydrodynamic radii, R_H_, calculated from dynamic light scattering data

| Sample | T | R_H_ / nm |
| --- | --- | --- |
| 0.01 wt% CaldA | 20 ^o^C | 113 ± 91 |
|  | 20 ^o^C cooled from 65 ^o^C | 113 ± 103 |
|  | 20 ^o^C cooled from boiled | 113 ± 94 |
|  | 65 ^o^C | 63 ± 73 |
| 0.01 wt% CaldA | 20 ^o^C | 113 ± 67 |
| 5x10^-3^ wt% CaldA  +50 mM 6-CF | 20 ^o^C | 113 ± 46 |
|  | 20 ^o^C cooled from boiled | 143 ± 64 |
| 0.01 wt% CaldA  +9.6 mM calcein | 20 ^o^C | 113 ± 86 |
|  | 20 ^o^C cooled from boiled | 143 ± 52 |

**SI Table S2.** Parameters extracted from the fitting of the SAXS data for 0.25 wt% solution of CaldA lipid shown in Fig. 1b.^a^

|  | **0.25 wt% CaldA** |
| --- | --- |
| *t* ± Δ*t* [Å] | 41.02 ± 0.58 |
| *ρ*_H_ [cm^-1^] | 2.07×10^-7^ |
| *σ*_H_ [Å] | 5.0 |
| *ρ*_C_ [cm^-1^] | -1.88 ×10^-7^ |
| *σ*_C_ [Å] | 5.0 |
| *D_i_* [Å] | 807 |
| *C* | 3.00×10^-3^ |

^a^ Data fitted using the software SASfit.^1-2^

**Key: Gaussian bilayer:** half layer thickness *t* (Gaussian polydispersity Δ*t*), scattering contrast of outer (headgroup) layers *ρ*_H_, and core (lipid chain) layer *ρ*_C_, Gaussian widths *σ*_C_ and *σ*_H_ of core and headgroup layers respectively, *D_i_* diameter (width) of layer system (when *D* >> *t* as here, it acts as a scaling parameter for the form factor). **Background:** constant background, *C*.

**SI Table S3.** MD simulations performed with membrane curvature indicated

| **System** | **Chain density** | **Curvature** |
| --- | --- | --- |
| A) ↑↑↑↑ | 500 chains in 7 × 7 nm = 10.2 chains nm^-2^ | Minimal |
| B) ↑↑↑↑ | 500 chains in 12 × 12 nm = 3.5 chains nm^-2^ | ✓ |
| C) ↑↑↑↑ | 400 chains in 12 × 12 nm = 2.8 chains nm^-2^ | ✓ |
| D) ↑↓↑↓ | 500 chains in 5 × 5 nm = 20 chains nm^-2^ | ✓ |
| E) ↑↓↑↓ | 300 chains in 5 × 5 nm = 12 chains nm^-2^ | ✓ |

**References**

(1)	Bressler, I.; Kohlbrecher, J.; Thünemann, A. F., SASfit: a tool for small-angle scattering data analysis using a library of analytical expressions. *J. Appl. Cryst.* **2015,** *48*, 1587-1598.

(2)	Kohlbrecher, J.; Bressler, I., Updates in SASfit for fitting analytical expressions and numerical models to small-angle scattering patterns. *J. Appl. Cryst.* **2022,** *55*, 1677-1688.
